# A309 THE DESCRIPTION OF NUTRITION COUNSELLING FOR IRRITABLE BOWEL SYNDROME IN THE LITERATURE IS CURRENTLY LACKING

**DOI:** 10.1093/jcag/gwad061.309

**Published:** 2024-02-14

**Authors:** M E Lau, L W Liu, C H Parker

**Affiliations:** University of Toronto, Toronto, ON, Canada; University of Toronto, Toronto, ON, Canada; University of Toronto, Toronto, ON, Canada

## Abstract

**Background:**

Dietary interventions are frequently recommended for the management of irritable bowel syndrome (IBS). Anecdotally, the nutrition counselling received by patients with IBS varies. In clinical practice, the optimal way to counsel these patients in regard to the application of dietary therapy remains unclear.

**Aims:**

The aim of this scoping review is to summarize how the nutrition counselling component of dietary interventions for IBS is currently described in the literature.

**Methods:**

A search of English language publications between January 1, 1994 and September 28th, 2023 was conducted using MEDLINE. Search results were limited to studies in humans and adults over age 18. The following combination of MeSH keywords was used in the search: irritable bowel syndrome AND diet food and nutrition OR counseling OR patient education OR practice guidelines OR nutrition therapy. Publications that involved providing instruction to patients to modify their diets were included. Titles and then abstracts were screened to determine if they potentially met inclusion criteria. Eligible publications were then reviewed in full. Those publications that met the inclusion criteria were included in this scoping review. Information regarding the description of nutrition counseling as a component of the dietary intervention was collected. Analysis is descriptive.

**Results:**

The search strategy identified 683 potential articles. After title review, 251 abstracts were obtained and reviewed producing 31 publications that were reviewed in full. All 31 publications were included in the scoping review. Nutrition counselling was mentioned in all 31 publications, but the description of the components of nutrition counselling varied (Figure 1). No publication included all important components of nutrition counselling in the description of the dietary intervention.

**Conclusions:**

Patients with IBS frequently report symptoms triggered by food. Counselling is an important component to the delivery of dietary interventions in IBS. However, the nutrition counselling components of these interventions for IBS is not well described in the literature in a reliable or standardized way. This limits the ability to provide these therapies to patients in an optimal way. Furthermore, the impact of different nutrition counselling strategies on the efficacy of dietary interventions remains unknown.

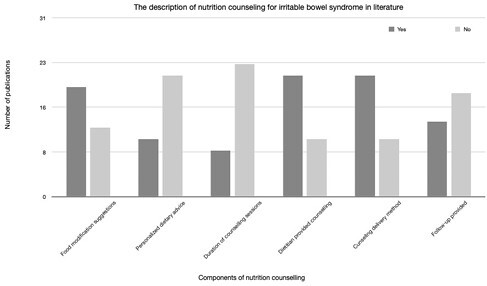

**Figure 1:** Results of Scoping Review.

**Funding Agencies:**

None

